# Modulation of endogenous plasmalogens by genetic ablation of lysoplasmalogenase (*Tmem86b*) in mice

**DOI:** 10.1016/j.jlr.2025.100808

**Published:** 2025-04-17

**Authors:** Sudip Paul, Pooranee Morgan, Gerard Pernes, Yvette Schooneveldt, Thy Duong, Natalie A. Mellett, Kevin Huynh, Andrew J. Murphy, Graeme I. Lancaster, Peter J. Meikle

**Affiliations:** 1 Metabolomics Laboratory, Baker Heart and Diabetes Institute, Melbourne, Victoria, Australia; 2 Faculty of Medicine, Nursing and Health Sciences, Monash University, Melbourne, Victoria, Australia; 3 Baker Department of Cardiovascular Research Translation and Implementation, La Trobe University, Bundoora, Victoria, Australia; 4 Haematopoesis and Leukocyte Biology Laboratory, Baker Heart and Diabetes Institute, Melbourne, Victoria, Australia; 5 Baker Department of Cardiometabolic Health, University of Melbourne, Parkville, Victoria, Australia

**Keywords:** glycerophospholipids, lipidomics, lipids, lysoplasmalogenase, phospholipids/metabolism, plasmalogens, plasmalogens/catabolism

## Abstract

Plasmalogens are a distinct subclass of glycerophospholipids that exhibit unique structural features, notably possessing a vinyl ether linkage at the sn1 position of the glycerol backbone. These specialized lipids play crucial roles in various biological functions. Although the biosynthetic pathway of plasmalogens has been well-characterized, their catabolism remains less studied. In this study, we investigated the impact of global and tissue-specific loss-of-function of a plasmalogen catabolizing enzyme, lysoplasmalogenase (TMEM86B), on circulatory and tissue lipidomes. We generated both global and hepatocyte-specific *Tmem86b* knockout mice using cre-loxP technology. Mice with homozygous global inactivation of *Tmem86b* (*Tmem86b* KO mice) were viable and did not display any marked phenotypic abnormalities. *Tmem86b* KO mice demonstrated significantly elevated levels of the plasmalogens, alkenylphosphatidylethanolamine (PE(P)), and alkenylphosphatidylcholine (PC(P)), as well as lysoplasmalogens, in the plasma, liver, and natural killer cells compared to their wild-type counterparts. The endogenous alkenyl chain composition of plasmalogens remained unaltered in *Tmem86b* KO mice. Consistent with the global knockout findings, hepatocyte-specific *Tmem86b* knockout mice also exhibited increased plasmalogen levels in the plasma and liver compared to their floxed control counterparts. Overall, our findings shed light on the role of *Tmem86b* in plasmalogen catabolism, demonstrating how its ablation leads to elevated plasmalogen levels in select tissues and cells. This study enhances our understanding of the regulatory mechanisms governing plasmalogen metabolism and highlights the potential of targeting *Tmem86b* to therapeutically raise plasmalogen levels.

Plasmalogens represent a class of glycerophospholipids characterized by a vinyl ether bond at the sn1 and an ester bond at the sn2 position of the glycerol backbone (1, 2). The vinyl ether-linked fatty alcohol at the sn1 position primarily consists of 16:0, 18:0, and 18:1 alkenyl groups, while the sn2 position is most often esterified with polyunsaturated fatty acids such as arachidonic acid (AA; 20:4) or docosahexaenoic acid (DHA; 22:6) (3). The predominant subclasses of plasmalogens in mammalian tissues are alkenylphosphatidylethanolamine (PE(P)) and alkenylphosphatidylcholine (PC(P)) (4).

Plasmalogens exist in different mammalian tissues in substantial amounts such that they can constitute up to 20% of the total phospholipid mass in humans (2). However, the tissue plasmalogen content is highly variable, where levels are high in brain, heart, lung, kidney, skeletal muscle, and certain immune cells but relatively low in liver (4, 5, 6). The low plasmalogen content in liver could be due to a number of factors, including transport of liver-generated plasmalogens to other tissues through lipoproteins, decreased synthesis of plasmalogens, or increased turnover of plasmalogens (7, 8). The intestine could also play an important role in distributing plasmalogens to other tissues through lipoproteins. While both hepatic and intestinal lipoproteins are possibly involved in plasmalogen transport, the precise contribution of these pathways to tissue plasmalogen levels remains unclear. While exact proportions are not definitively quantified, hepatic-derived lipoproteins (e.g., HDL, LDL) are considered the primary carriers of plasmalogens to tissues, given the liver’s central role in lipid metabolism (9). In contrast, intestinal-derived chylomicrons likely play a secondary role, primarily influencing postprandial lipid transport, containing diet-derived plasmalogens (10).

The primary site of plasmalogen synthesis remains a topic of debate, with ongoing controversy regarding whether plasmalogens are primarily synthesized in the liver and subsequently distributed throughout the body or whether they are produced locally within different organs. The liver exhibits minimal to no expression of fatty acyl-CoA reductase 1 or 2 (11), key enzymes required for plasmalogen biosynthesis, resulting in low hepatic plasmalogen levels. Moreover, hepatocyte-specific peroxisomal defects do not appear to affect plasmalogen levels in other tissues (12), which challenges the proposed role of the liver in supplying plasmalogens to other tissues. Instead, these findings suggest that plasmalogens may be synthesized locally within various tissues, with each organ possessing the necessary enzymatic machinery to regulate its own plasmalogen levels.

Plasmalogens are important structural constituents of the biological membranes of animals and certain anaerobic bacteria, and have several well-described functions, including regulating membrane dynamics (13) and vesicular cholesterol transport and homeostasis (14, 15, 16, 17). One of the most interesting features of plasmalogens is their endogenous antioxidant activity, which is mostly due to the vinyl ether bond, which can scavenge reactive oxygen species and thereby protect other biomolecules from oxidative damage (1, 18). They have also been found to increase the gene expression of multiple antioxidant enzymes to protect against chemically induced cytotoxicity and lipid peroxidation in cultured hepatocytes (19). Plasmalogen derivatives such as polyunsaturated fatty acids (AA or DHA) and lysoplasmalogens can act as lipid mediators for multiple cellular signaling activities (20). Plasmalogens have also been found to be important for phagocytosis of macrophages (21), lipid droplet formation (22), and development and function of neuromuscular junctions (23). Furthermore, they play vital roles in mediating immune responses (24, 25) and mitochondrial fission to regulate adipose tissue thermogenesis (26) and protecting neuronal cells against cell death (27) and inflammation (28, 29). All of these are suggestive of a critical role played by plasmalogens in maintaining cellular homeostasis.

Lipid profiling of multiple population cohorts has identified plasmalogen deficiency in neurodegenerative and cardiometabolic diseases. For instance, reduced plasmalogens were found in the affected brain regions of patients with Alzheimer’s disease (AD), with the extent of reduction correlated to disease severity (30, 31). In another study, circulating plasmalogen deficiency in AD patients was found to be correlated with the severity of dementia (32). Moreover, decreased levels of plasmalogens are associated with aging and obesity (33, 34) as well as prediabetes and type 2 diabetes (35). In patients with coronary artery disease, lower plasmalogen levels were observed in stable disease (relative to healthy control individuals) and further decreased levels in unstable disease (relative to stable disease) (36).

While plasmalogen anabolism is well defined, its catabolism has been less studied. During catabolism, plasmalogens are deacylated by the action of a calcium-independent phospholipase A2 enzyme (iPLA2) to produce lysoplasmalogens (37, 38) (Fig. 1). However, cytochrome C has also been shown to act as a plasmalogenase under certain circumstances (39). The amount of lysoplasmalogens in cells is tightly regulated (40) either by reacylation into plasmalogens through a coenzyme A-independent transacylase (41) or by degradation into fatty aldehydes and glycerophospholipids by an alkenyl ether hydrolase commonly known as lysoplasmalogenase (Fig. 1). Lysoplasmalogenase (EC 3.3.2.2 and EC 3.3.2.5) is a microsomal transmembrane enzyme highly specific for lysoplasmalogens and has no activity against plasmalogens (42, 43, 44, 45, 46). The enzyme has been identified and studied in microsomes of the rat liver (44, 45, 47) and small intestinal mucosa (46), where its specific activities are high. The enzyme has also been found to exist in brain microsomes; however, its activity is very low in the brain, only 1/700^th^ of liver activity (42, 43). The gene encoding lysoplasmalogenase, *Tmem86b* has been found to be expressed differentially across different mouse tissues (48). *Tmem86b* expression generally has an inverse association with plasmalogen abundance; for example, liver, which has high levels of *Tmem86b* expression, has low levels of plasmalogens (34, 48). Moreover, overexpression of *TMEM86B* in HEK 293T cells led to about 20% reduction of cellular plasmalogen levels (48). *TMEM86B* is a relatively small gene, with only a handful of predicted loss-of-function mutations, all of which are extremely rare (https://gnomad.broadinstitute.org/gene/ENSG00000180089). The most common is a frameshift mutation observed in only 67 heterozygous individuals (49). However, a recent lipidomic genome-wide association study (50) identified a missense mutation in *TMEM86B* (https://metabolomics.baker.edu.au/pheweb_meta/gene/TMEM86B; 19-55738634-T-C) that leads to increased plasma PE(P) species levels when arginine at position 199 is replaced with histidine. Further to this, a close homolog of TMEM86B, TMEM86A, has recently been shown to exhibit lysoplasmalogenase activity in adipocytes (51). These observations indicate that lysoplasmalogenases play an important role in regulating endogenous plasmalogen content. To extend our current knowledge about the role of lysoplasmalogenase in regulating endogenous plasmalogen levels, here we generated *Tmem86b* knockout mice and characterized their plasma and tissue lipidomes.

**Fig. 1:**
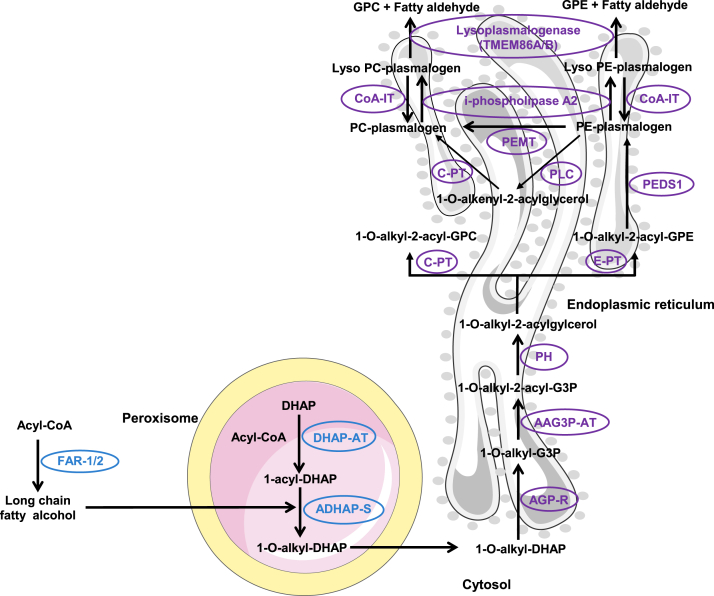
Biosynthetic and catabolic pathways of plasmalogens. AAG3P-AT, alkyl/acyl-glycero-3-phosphate acyltransferase; ADHAP-S, alkyl DHAP synthase (Gene: *AGPS*); AGP-R, acylglycerone phosphate reductase (Gene: *DHRS7B*); CoA, coenzyme A; CoA-IT, coenzyme A-independent transacylase; C-PT, choline phosphotransferase (Gene: *CEPT1*); DHAP, dihydroxyacetone phosphate; DHAP-AT, DHAP acyltransferase (Gene: *GNPAT*); E-PT, ethanolamine phosphotransferase or selenoprotein 1 (Gene: *EPT1*); FAR-1/2, fatty acyl-CoA reductase 1 or 2 (Gene: *FAR1/2*; ER, endoplasmic reticulum; GPC, glycerophosphocholine; GPE, glycerophosphoethanolamine; i-phospholipase A2, calcium independent phospholipase A2; PC, phosphatidylcholine; PE, phosphatidylethanolamine; PEDS1, plasmanylethanolamine desaturase 1 (Gene: *PEDS1*); PEMT, phosphatidylethanolamine N-methyltransferase (Gene: *PEMT*); PH, phosphohydrolase; PLC, phospholipase C.

## Methods and Materials

### Generation of global and hepatocyte-specific *Tmem86b* knockout mice

Targeted embryonic stem (ES) cells containing a gene-trap cassette consisting of both flippase-flippase recognition target (FLP-FRT) sites and locus of X-over P1 (loxP) sites-flanking exon 3 of *Tmem86b* allele were obtained from the European Conditional Mouse Mutagenesis (EUCOMM) Program (clone identification HEPD0809_6_F02). This allele is referred to as “targeted mutation 1a” (tm1a) and is a non-expressable form due to alternative splicing of the target gene by the trapping cassette (52). Microinjection of ES cells in C57BL/6N embryos and subsequent generation of mice bearing the tm1a allele were performed by the Monash Genome Modification Platform. Mice bearing one copy of the tm1a allele (*Tmem86b*^*tm1a/+*^; heterozygous knockout) were imported to Alfred Medical Research and Education Precinct Animal Centre and bred to create mice with all three possible genotypes (*Tmem86b*^*tm1a/tm1a*^; homozygous knockout, *Tmem86b*^*tm1a/+*^ and *Tmem86b*^*+/+*^; wild-type). Floxed *Tmem86b* mice (*Tmem86b*^*flox/flox*^) were further generated by crossing heterozygous tm1a mice with flippase recombinase (FLPeR)-expressing transgenic mice (Monash Animal Research Platform, Monash University, Melbourne, Australia) to remove the flippase recombinase target (FRT) site-flanked selection cassette and restore the expression of *Tmem86b*. *Tmem86b*^*flox/flox*^ mice were subsequently crossed with transgenic mice expressing Cre recombinase (Jackson Laboratory; stock no: 018,961) under control of the albumin promoter (Alb-Cre) to generate hepatocyte-specific *Tmem86b* knockout (*Tmem86b*^*flox/flox*^*; Alb-Cre*) mice. All the mouse lines used in this study were on a C57BL/6N background.

### Genotyping of animals

Mouse genotypes were determined from tail samples by a commercial vendor (Transnetyx), using TaqMan® real-time polymerase chain reaction (PCR) assays with specific primers and probes designed for each allele. Transnetyx employs a real-time PCR-based system for genotyping. Their TaqMan® real-time PCR assays target specific deoxyribonucleic acid (DNA) sequences, amplifying millions of copies of the target molecule and generating a real-time fluorescent readout throughout the reaction. These assays are designed to bind to the target DNA in a 5′ to 3′ orientation, with reaction specificity ensured by a fluorescent-labelled probe. Unlike methods that differentiate based on fragment size or length, this system relies on the presence of specific genomic DNA sequences for genotyping. For genotyping the wild-type (WT) chromosome 13, Transnetyx used two reported probes, whereas only one probe was employed for other alleles (WT *Tmem86b*, *tm1a*, floxed *Tmem86b*, and *Alb-Cre*). This approach follows the competitive allele-specific TaqMan® PCR strategy, which enhances primer specificity by incorporating an additional reporter probe to compete against the alternative allele. The primer and probe sequences used for genotyping the mice are listed in Supplemental Table S1.

### Animal experimentation

Mice were housed in the Alfred Medical Research and Education Precinct Animal Centre under a 12-h light-dark cycle, temperature-controlled environment. All mice were fed a standard irradiated mouse diet (Specialty Feeds). For characterization, 12-week-old male global and hepatocyte-specific *Tmem86b* knockout mice and their littermate controls were fasted for 6 h and then humanely killed either with sodium pentobarbitone (Lethabarb; 200 mg/kg, i.p.) or CO_2_ asphyxiation. Whole blood was drawn from the animals by cardiac puncture, and different organs (liver, brown and subcutaneous adipose tissues, heart, small intestine, and brain) were immediately dissected. Whole blood was collected in EDTA-containing tubes, and the plasma was separated by centrifugation at 3,000 rpm for 10 min and aliquoted, and stored at −80°C. Tissue samples were snap frozen in liquid nitrogen and stored at −80°C until further use. All animal experimentations were conducted in accordance with the regulatory standards of the National Medical and Health Research Council of Australia and were approved by the Alfred Research Alliance Animal Ethics Committee (E/1840/2018/B).

### Biochemical analyses

Fasting blood glucose level was measured from the tail blood of live animals using a glucometer (Accu-Check Performa; Roche). The levels of plasma triglycerides, free cholesterol and high-density lipoprotein-cholesterol (HDL-C) and plasma activity of the enzyme alanine aminotransferase (ALT) and aspartate aminotransferase (AST) were measured using commercially available kits on a COBAS Integra 400 Plus blood chemistry analyzer (Roche Diagnostics) following standard guidelines.

### RNA isolation and reverse transcription–quantitative PCR

Total RNA was isolated using a commercially available kit (RNeasy Mini Kit, Qiagen) and RNA quality and quantity was determined using a Nanodrop spectrophotometer (Thermo Scientific). Complementary DNA was synthesized by reverse transcription using a High-Capacity cDNA Reverse Transcription Kit (Life Technologies, Thermo Fisher Scientific) according to the manufacturer’s recommendations. Quantitative PCR was performed using Taqman probes [assay IDs: *Hprt* (Mm00446968_m1), *Tmem86b* (Mm01330963_m1), *Tmem86a* (Mm01206370_g1)] and amplified on an Applied Biosystems Quant7 real time PCR instrument (Life Technologies). Target mRNA expression was normalized to hypoxanthine phosphoribosyltransferase (*Hprt*) expression and expressed as a relative value using the 2 ^−ΔΔCt^ method of quantification.

### Isolation of immune cells from bone marrow

The immune cells analyzed in this study were collected from bone marrow (BM). Following humane killing by CO_2_ asphyxiation, hind limb bones were collected, and BM was harvested by flushing bones with RPMI media. Red blood cells were lysed, and then white blood cells were stained with the antibody cocktails outlined below (Table 1) for cell-specific surface markers and incubated for 30 min on ice in the dark. Antibodies were used at a 1:400 dilution unless stated otherwise. Staining was stopped with FACS buffer, and cells were subsequently washed and filtered through a 35 μm strainer prior to sorting.

**Table 1:** Antibodies and sorting panels used to purify murine immune cells

Immune cell type	Antibodies used in sorting panels
B cells	L/D CD45^+^ CD3^-^ CD19^+^
Natural killer cells	L/D CD45^+^ CD3^-^ NK1.1^+^
T cells	L/D CD45^+^ CD3^+^
Monocytes	L/D CD45^+^ CD115^+^
Neutrophils	L/D CD45^+^ CD115^-^ Gr1^+^

Fluorescence-activated cell sorting (FACS) was performed at the Alfred Research Alliance (ARA) flow cytometry core facility (ARAFlowCore). Individual cell populations were sorted using BD FACSAria Fusion (BD Biosciences). All gating strategies were first set up based on forward scatter area versus side scatter area, forward scatter height versus forward scatter area (doublet exclusion), and side scatter area versus viability dye (viable cell isolation). A sorted event threshold was set to 60,000 cells, and cells were sorted according to the expression of the specific surface markers detailed in the preceding table. Following isolation, cells were washed with phosphate-buffered saline PBS w/o Ca^2+^ and Mg^2+^ (PBS; pH 7.6) and stored at −80°C.

### Tissue homogenization for lipidomic analysis

Approximately 40–50 mg of snap frozen tissue samples (liver, visceral, subcutaneous and brown adipose tissues, heart, small intestine and brain) were homogenized in 500 μl of ice-cold PBS using a probe homogenizer (Bio-Gen Pro200, PRO Scientific) for 10 s and then sonicated with an ultrasonic probe sonicator (Misonix S-4000, Thermo Fisher Scientific) for 15 s at amplitude 20. Protein content of the homogenates and cell lysates was determined using a Pierce® bicinchoninic acid (BCA) assay kit (Thermo Fisher Scientific) following the manufacturer’s guidelines. Homogenates were made up to a stock protein concentration of 5 mg/ml and 10 μl aliquots from the stock containing 50 μg of protein were subsequently used for lipid extraction.

### Lipid extraction

Lipids were extracted by a chloroform: methanol (2:1) method as described previously (29). Briefly, 10 μl of plasma, homogenized tissue, or immune cell lysate (in PBS) was mixed with internal standards (Supplemental Table S2), and the lipids were then extracted using 200 μl of chloroform: methanol (2:1). The extracted lipids were then dried under a stream of nitrogen at 40°C and finally reconstituted with 50 μl of water saturated butanol and 50 μl methanol containing 10 mmol/l ammonium formate. The lipid extracts were stored at −80°C until further analysis.

### Liquid chromatography electrospray ionization tandem mass spectrometry

Lipidomic analysis was performed using an Agilent 1,290 ultra-high performance liquid chromatography system combined with either an Agilent 6495C triple quadrupole mass spectrometer (Agilent Technologies) or 4000 QTRAP mass spectrometer (AB SCIEX). Liquid chromatography separation was performed on a ZORBAX eclipse plus C18 column (2.1 × 100 mm, 1.8 μm, Agilent Technologies) or Poroshell C18 column (2.1 × 100 mm, Agilent Technologies). The details of the methods and chromatography gradients have been described previously (33, 53). The solvent system consisted of solvent A: 50% water/30% acetonitrile/20% isopropanol (v/v/v) containing 10 mM ammonium formate and solvent B: 1% water/9% acetonitrile/90% isopropanol (v/v/v) containing 10 mM ammonium formate.

The fragmentation patterns for the tandem mass spectrometry of each lipid class are provided in Supplemental Table S2. The resultant chromatograms were analyzed either by Masshunter Quant Analysis v10.0 (Agilent Technologies) or Multiquant v1.2 software (AB SCIEX, USA). The relative concentrations of individual lipid species were calculated by taking a ratio of the peak area of each lipid species to the peak area of the corresponding internal standard (Supplemental Table S2). The ratio was then multiplied by the amount of corresponding internal standard added into the sample and response factors were applied, where these are known. The concentrations of lipid classes were calculated by summing the concentrations of individual species within each class.

### Statistical analysis

The concentrations of lipid species/classes were normalized to phosphatidylcholine (PC) concentration in plasma and different tissues/cells to allow a direct comparison of the relative lipid levels between the sample types. The mean differences in lipid concentrations between animal groups were compared using Student’s *t* test (for 2 groups) or one-way ANOVA followed by Tukey’s honest significant difference (HSD) test (for more than 2 groups). The *P* values were adjusted for multiple comparisons using the Benjamini-Hochberg method unless otherwise stated, and a corrected *P* value less than 0.05 was considered statistically significant.

## Results

### *Tmem86b* expression in murine tissues

**Fig. 2:**
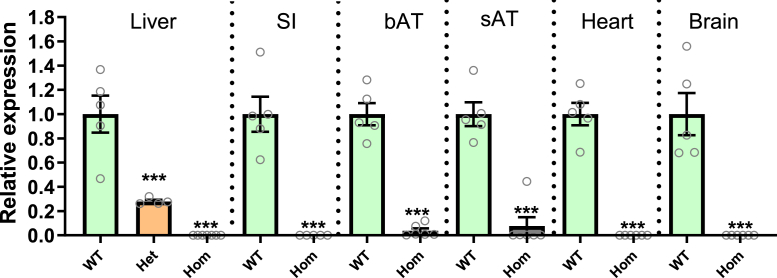
*Tmem86b* expression in wild-type and global *Tmem86b* knockout mice. mRNA expression levels of *Tmem86b* were normalized to hypoxanthine phosphoribosyltransferase (*Hprt*) expression and presented as mean ± SEM (12-week-old male mice, n = 4–7/group, each circle represents individual mouse data) relative to the wild-type (WT) group. The mean differences between the groups (wild-type and knockout) were analyzed using one-way ANOVA, followed by Tukey’s HSD *post hoc* test or Student *t* test; ∗∗∗ indicates *P* < 0.001 relative to the wild-type group. bAT, brown adipose tissue; Het, Heterozygous *Tmem86b* knockout; Hom, Homozygous *Tmem86b* knockout; sAT, subcutaneous adipose tissue; SI, small intestine.

We first evaluated the relative expression of *Tmem86b* across multiple mouse tissues. The expression of *Tmem86b* was largely confined to the liver and small intestine, with minimal levels detected in other tissues analyzed (Supplemental Fig. S1). Additionally, we confirmed that *Tmem86b* predominates over *Tmem86a* in the liver and small intestine (Supplemental Fig. S2A). As anticipated, *Tmem86b* expression was completely abolished in all tissues of homozygous *Tmem86b* KO (*Tmem86b* KO) mice (Fig. 2).

### Effects of global ablation of *Tmem86b* on plasma and tissue plasmalogen levels

**Fig. 3:**
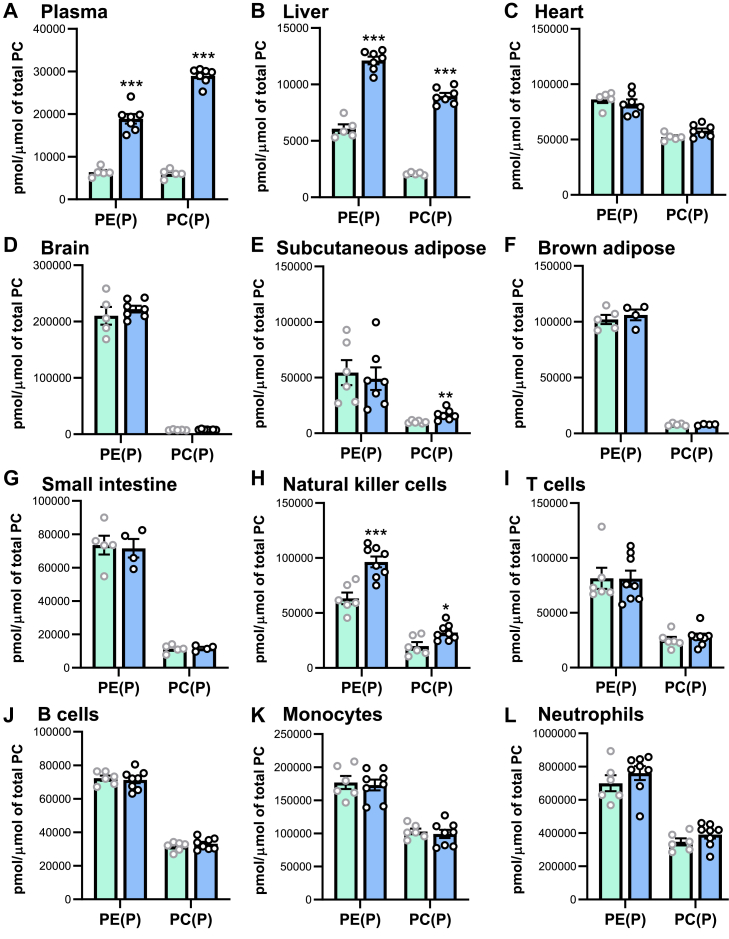
Plasmalogen levels in plasma, tissues, and immune cells of wild-type and global *Tmem86b* knockout mice. Concentrations of alkenylphosphatidylethanolamine (PE plasmalogen or PE(P)) and alkenylphosphatidylcholine (PC plasmalogen or PC(P)) were normalized to phosphatidylcholine (PC) in plasma (A), tissues (B–G) and bone marrow derived immune cells (H–L) of wild-type (cyan bar) and homozygous knockout (sky blue bar) mice. Data are presented as mean ± SEM (12-week-old male mice, n = 4–8/group, each circle represents individual mouse data). Student *t* test was performed to analyze the mean differences between the groups; ∗indicates *P* < 0.05, ∗∗ indicates *P* < 0.01, and ∗∗∗ indicates *P* < 0.001 relative to the wild-type group.

To determine whether *Tmem86b* KO leads to an enrichment of endogenous plasmalogens, we analyzed plasmalogen levels in plasma and different tissues. *Tmem86b* deletion resulted in a substantial increase in plasma plasmalogen levels (Fig. 3A). Compared with WT mice, *Tmem86b* KO mice had 3- and 5-fold higher plasma PE(P) and PC(P) levels, respectively (*P* < 0.001) (Fig. 3A and Supplemental Table S3). Higher total plasmalogen levels in the plasma of KO mice were due to increases in multiple plasmalogen species; 43 out of the 44 measured plasma PE(P) species were significantly higher in KO mice compared to WT mice (Supplemental Table S4). Furthermore, *Tmem86b* KO mice contained significantly higher levels of all 26 different PC(P) species measured when compared with WT mice (Supplemental Table S4).

Consistent with high *Tmem86b* expression in the liver, plasmalogen levels were likewise markedly elevated in the liver of *Tmem86b* KO mice (Fig. 3B and Supplemental Tables S5 and S6), while tissues with low *Tmem86b* expression (heart, brain, subcutaneous, and brown adipose tissues) were largely unaffected in these mice (Fig. 3C–F and Supplemental Tables S7–S14). However, in subcutaneous adipose tissue, the level of PC(P) was slightly, yet significantly higher in KO mice compared to WT mice (Fig. 3E). Given that *Tmem86b* expression was highest in the small intestine, we were somewhat surprised to observe that plasmalogen levels in the small intestine were unaffected by *Tmem86b* deletion (Fig. 3G, and Supplemental Tables S15 and S16).

In liver, the total levels of PE(P) and PC(P) were 2 and 4-fold higher, respectively, in *Tmem86b* KO mice compared with WT mice (*P* < 0.001) (Fig. 3B and Supplemental Table S5). Twenty nine out of 55 measured molecular PE(P) species were significantly increased (Supplemental Table S6). We particularly noted that the concentrations of PE(P) species with 22:4 and 22:5n3 fatty acids at the sn2 position were not significantly different between KO and WT mice (Supplemental Table S6). In addition to the remarkable changes in PE(P) species, the concentrations of 21 (among 23 measured) PC(P) species were significantly higher in KO mice compared with WT mice (Supplemental Table S6).

We have previously shown that plasmalogen levels vary markedly between different immune cell types (4) and therefore we next examined the impact of *Tmem86b* deletion on plasmalogen levels in various immune cells. While plasmalogen levels were markedly increased in natural killer (NK) cells (Fig. 3H, and Supplemental Tables S17 and S18) from *Tmem86b* KO mice compared to WT mice, they remained largely unaffected in T cells, B cells, monocytes, and neutrophils (Fig. 3I–L and Supplemental Tables S19–S26).

### Effects of global ablation of *Tmem86b* on plasmalogen composition in liver, plasma, and natural killer cells

The plasmalogen pool in plasma or tissue comprises multiple plasmalogen species with different alkenyl chains at the sn1 position and fatty acyl chains at the sn2 position. Here, we examined the impact of *Tmem86b* knockout on the alkenyl and acyl chain composition within the enriched plasmalogen pool across plasma, liver, and NK cells, as variations in these compositions of plasmalogens may influence their biological activities. For instance, the length and unsaturation of the alkenyl chains at the sn1 position of plasmalogens may influence their antioxidant properties (54). Additionally, the fatty acid composition at the sn2 position can influence antioxidant properties of plasmalogens (54) as well as impact membrane fluidity and integrity (55).

**Fig. 4:**
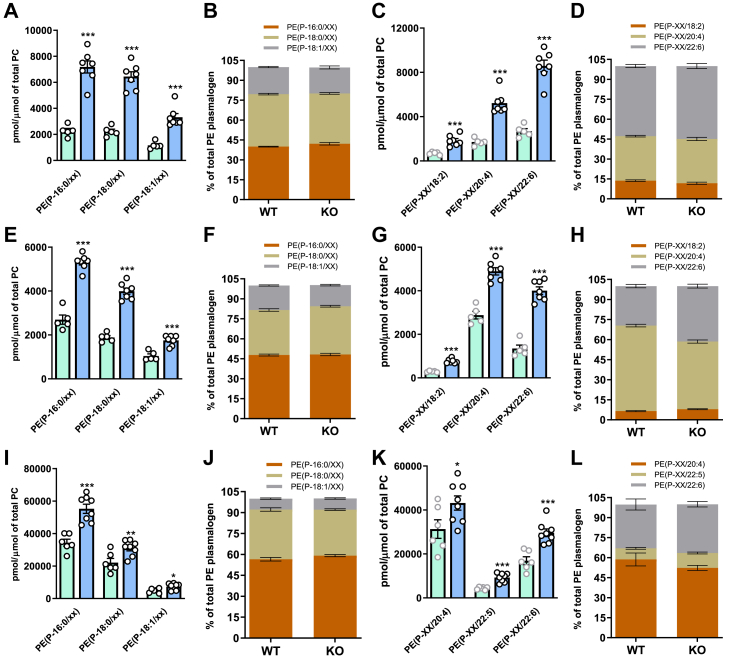
Plasmalogen composition in plasma, liver and natural killer cells of global *Tmem86b* knockout mice. Concentrations of alkenyl chain containing alkenylphosphatidylethanolamine (PE plasmalogen or PE(P)) were calculated by summing the concentrations of the individual species containing each alkenyl chain type. These data are presented as concentrations normalized to phosphatidylcholine in plasma (A), liver (E) and natural killer cells (I) and as proportions of different alkenyl chain containing PE(P) in plasma (B), liver (F) and natural killer cells (J). Concentrations of different acyl chain containing PE(P) were calculated in the same manner and are expressed as concentrations normalized to phosphatidylcholine in plasma (C), liver (G) and natural killer cells (K) and as proportions of different acyl chain containing PE(P) in plasma (D), liver (H) and natural killer cells (L). Genotypes are shown as: wild-type (WT, cyan bar) and homozygous knockout (KO; sky blue bar) mice. Data are presented as mean ± SEM (12 week old male mice, n = 5–7/group, each circle represents individual mouse data). Student *t* test was performed to analyse the mean differences between the groups; ∗ indicates *P* < 0.05, ∗∗ indicates *P* < 0.01 and ∗∗∗ indicates *P* < 0.001 relative to the wild-type group.

In plasma, the levels of various alkenyl chain-containing PE plasmalogens, specifically P-16:0/XX, P-18:0/XX, and P-18:1/XX, were significantly higher in *Tmem86b* KO mice compared to WT mice (Fig. 4A); however alkenyl chain composition of PE plasmalogens was largely unaffected (Fig. 4B). Likewise, while the overall levels of PE(P) with various fatty acids were elevated in KO mice (Fig. 4C), the relative proportions of different fatty acid-containing plasma PE(P) remained consistent between KO and WT mice (Fig. 4D). Similar effects were seen in both the liver (Fig. 4E–H) and NK cells (Fig. 4I–L). However, in the liver of KO mice compared to WT mice, there was a noticeable increase in the proportion of 22:6-containing PE(P) and a decrease in 20:4-containing PE(P) (Fig. 4H).

### Effects of global ablation of *Tmem86b* on other lipid classes in liver, plasma, and natural killer cells

**Fig. 5:**
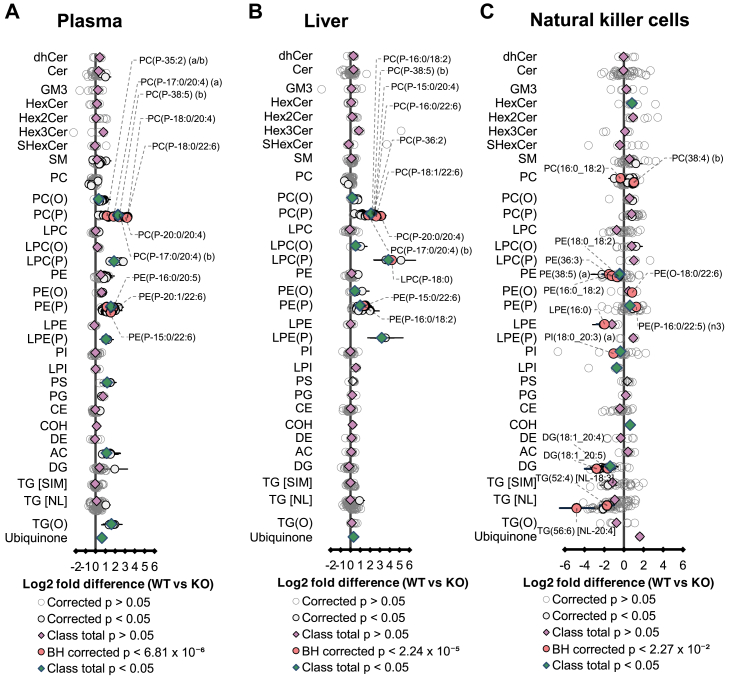
Lipidome of plasma, liver, and natural killer cells of wild-type and global *Tmem86b* knockout mice. Fold differences in plasma (A), liver (B), and natural killer cells (C) lipid concentrations between wild-type (WT) and homozygous *Tmem86b* knockout (KO) mice (12-week-old male; n = 5–7/group) were analyzed by Student *t* test. Data are presented as Log2 fold difference of the mean; whiskers represent 95% confidence intervals. *P*-values were corrected for multiple comparisons using the Benjamini and Hochberg method. CE, cholesteryl ester; Cer, ceramide; COH, cholesterol; DE, dehydrocholesteryl ester; DG, diacylglycerol; dhCer, dihydroceramide; GM3, GM3 ganglioside; HexCer, monohexosylceramide; Hex2Cer, dihexosylceramide; Hex3Cer, trihexosylceramide; LPC, lysophosphatidylcholine; LPC(O), lysoalkylphosphatidylcholine; LPC(P), lysoalkenyl-phosphatidylcholine; LPE, lysophosphatidylethanolamine; LPE(P), lysoalkenylphosphatidylethanolamine; LPI, lysophosphatidylinositol; PC, phosphatidylcholine; PC(O), alkylphosphatidylcholine; PC(P), alkenylphosphatidylcholine; PE, phosphatidylethanolamine; PE(O), alkylphosphatidylethanolamine; PE(P), alkenylphosphatidylethanolamine; PI, phosphatidylinositol; PG, phosphatidylglycerol; PS, phosphatidylserine; SM, sphingomyelin; TG [NL], triacylglycerol (analyzed by neutral loss scan); TG [SIM], triacylglycerol (analyzed by single ion monitoring); TG(O), monoalkyl-diacylglycerol.

Given the pronounced impact of global *Tmem86b* deletion on plasmalogen levels in plasma and selected tissues, we sought to investigate how other lipid classes were affected in these tissues. In addition to the elevated plasmalogen contents in plasma of *Tmem86b* KO mice, we identified significantly elevated concentrations of alkylphosphatidylcholine (PC(O)) (26%), lysoalkenylphosphatidylcholine (LPC(P)) (269%), lysoalkenylphosphatidylethanolamine (LPE(P)) (112%), phosphatidylserine (PS) (139%), acylcarnitine (AC) (114%), monoalkyl-diacylglycerols (TG(O)) (225%), and ubiquinone (61%) in *Tmem86b* KO mice compared to WT mice (Fig. 5A).

Moreover, the livers of KO mice exhibited significantly increased levels of PC(O) (14%), lyso PC(O) (44%), LPC(P) (1271%), PE(O) (35%), LPE(P) (670%), and ubiquinone (25%) relative to WT mice (Fig. 5B). In NK cells of KO mice, alterations in non-plasmalogen lipid classes were also observed, with significantly higher levels of monohexosylceramides (HexCer) (75%) and cholesterol (COH) (52%) alongside reduced levels of PE (26%), phosphatidylinositol (PI) (21%), lysophosphatidylinositol (LPI) (41%), and diacylglycerol (DG) (62%) compared to WT controls (Fig. 5C).

These findings indicate that *Tmem86b* deletion, accompanied by changes in plasmalogen and lysoplasmalogen levels, exerts downstream effects on several other lipid classes. However, overall, the magnitude of these alterations in non-plasmalogen lipid classes was relatively less pronounced compared to the changes observed in plasmalogen and lysoplasmalogen lipid classes.

### Effects of global ablation of *Tmem86b* on basic metabolic parameters

Finally, we wanted to assess how the changes in plasmalogen levels impacted markers of systemic lipid metabolism in the *Tmem86b* KO mice. Firstly, we did not observe any gross morphological tissue abnormalities in the KO mice. There was no difference in body weight between *Tmem86b* KO mice and their WT littermates (Table 2). Furthermore, there was no significant difference between genotypes in liver weight, plasma levels of fasting blood glucose, triglycerides, free cholesterol, HDL-C, or plasma ALT and AST activities (Table 2). These findings demonstrate that *Tmem86b* KO mice have relatively normal basal metabolic profiles.

**Table 2:** Baseline characteristics of wild-type and global *Tmem86b* knockout mice

Parameter	WT	Het KO	Hom KO
Body weight (g)	26.02 ± 0.41	26.88 ± 0.54	27.43 ± 0.66
Liver weight (g)	1.24 ± 0.02	1.22 ± 0.04	1.20 ± 0.04
FBG (mmol/L)	10.36 ± 0.66	9.98 ± 0.45	9.10 ± 0.52
TG (mmol/L)	0.64 ± 0.08	0.49 ± 0.09	0.85 ± 0.07
COH (mmol/L)	1.71 ± 0.08	1.69 ± 0.15	1.51 ± 0.10
HDL-C (mmol/L)	1.58 ± 0.07	1.53 ± 0.12	1.31 ± 0.11
ALT (U/L)	17.80 ± 1.40	20.02 ± 3.35	20.40 ± 1.25
AST (U/L)	92.75 ± 20.81	79.63 ± 9.00	118.81 ± 22.02

Data are presented as mean ± SEM (12-week-old male mice, n = 4–7/group); ALT, alanine aminotransferase; AST, aspartate aminotransferase; COH, free cholesterol; FBG, fasting blood glucose; HDL-C, high-density lipoprotein cholesterol; Het KO, heterozygous knockout; Hom KO, homozygous knockout; TG, triglyceride; WT, wild-type.

The mean differences between the groups (wild-type and knockout) were analyzed using one way ANOVA followed by Tukey’s HSD *post hoc* test.

### Hepatic and plasma lipidomes of hepatocyte-specific *Tmem86b* knockout mice

**Fig. 6:**
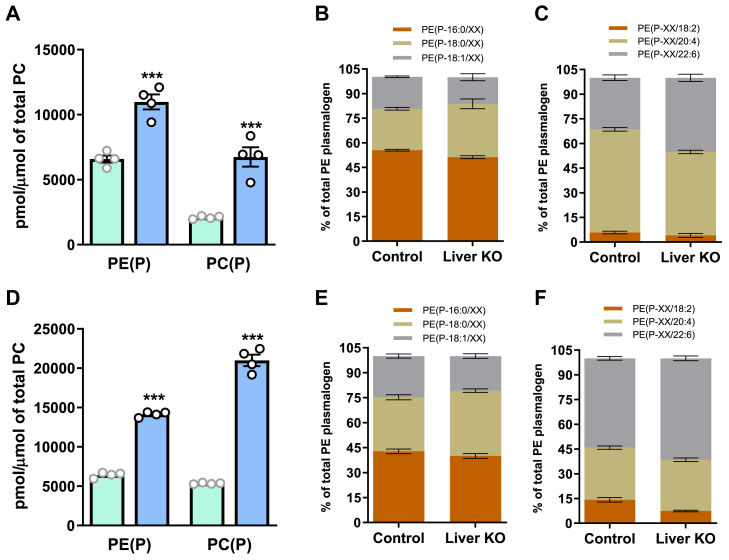
Plasmalogen levels and composition in liver and plasma of hepatocyte-specific *Tmem86b* knockout mice. Concentrations of alkenylphosphatidylethanolamine (PE plasmalogen or PE(P)) and alkenylphosphatidylcholine (PC plasmalogen or PC(P)) were normalized to phosphatidylcholine (PC) in liver (A) and plasma (D) of floxed control (cyan bar) and hepatocyte-specific *Tmem86b* knockout (Liver KO) (sky blue bar) mice. Concentrations of different alkenyl chain containing PE(P)) were calculated by summing the concentrations of the individual species containing each alkenyl chain type. These data are presented as proportions of different alkenyl chain-containing PE(P) in liver (B) and plasma (E). Concentrations of different acyl chain containing PE(P) were calculated in the same manner and are expressed as proportions of different acyl chain containing PE(P) in liver (C) and plasma (F). Data are presented as mean ± SEM (12-week-old male mice, n = 4/group, each circle represents individual mouse data). Student *t* test was performed to analyze the mean differences between the groups; ∗∗∗ indicates *P* < 0.001.

Considering the prominent impacts observed in plasma and hepatic plasmalogens by global *Tmem86b* ablation, we aimed to understand if changes in hepatic plasmalogens drove subsequent plasma plasmalogen alterations. To this end, we generated floxed *Tmem86b* (FC) mice and subsequently crossed these with Albumin-Cre mice to create hepatocyte-specific *Tmem86b* knockout (HKO) mice. HKO mice did not have any *Tmem86b* expression in the liver but had normal *Tmem86b* expression (relative to FC mice) in other tissues examined (Supplemental Fig. S3). Similar to global KO mice, we observed significantly higher levels of hepatic plasmalogens in HKO mice. The HKO mice had 2- and 3-fold higher PE(P) and PC(P) levels, respectively (*P* < 0.001) compared with FC mice (Fig. 6A, and Supplemental Tables S27 and S28). While the overall concentration of hepatic PE(P) was elevated in HKO mice, the alkenyl chain composition of PE(P) was consistent relative to FC mice (Fig. 6B) but there was a notable shift in fatty acyl composition, with decreased 20:4 PE(P) and increased 22:6 PE(P) (Fig. 6C). Similar to global KO mice, HKO mice also had significantly higher plasma plasmalogens compared with FC mice. HKO mice contained 2- and 4-fold higher plasma PE(P) and PC(P), respectively relative to FC mice (*P* < 0.001) (Fig. 6D, and Supplemental Tables S29 and S30). The alkenyl chain composition of plasma PE(P) was conserved in HKO mice as FC mice (Fig. 6E); however, the acyl chain composition was altered, with a significant increase in 22:6 PE(P) and a decrease in 18:2 PE(P) compared to FC mice (Fig. 6F).

## Discussion

Plasmalogen catabolism is initiated either by deacylation, catalyzed by plasmalogen-specific calcium independent phospholipase A2 (2, 56), or by oxidative cleavage mediated by cytochrome C (39). The lysoplasmalogens generated through the deacylation reaction may subsequently undergo further degradation via lysoplasmalogenase (TMEM86A or TMEM86B) (48, 51) or be reacylated into plasmalogens through the action of coenzyme-A independent transacylase (57) (Fig. 1). In this study, we generated KO mice with a targeted disruption of *Tmem86b* to evaluate the impact of this genetic ablation on circulatory and tissue lipidomes. Our findings reveal that *Tmem86b* deletion led to significant increases in plasmalogens in the liver, plasma, and NK cells, providing further understanding of the role of TMEM86B in plasmalogen catabolism.

*Tmem86b* KO mice appeared to be healthy without any noticeable metabolic effects. Notably, these KO mice exhibited significantly elevated levels of lysoplasmalogens and plasmalogens in plasma and liver compared to WT mice. While research on the distinct biological functions of lysoplasmalogens and plasmalogens is lacking, some reports indicate potential toxic effects of lysoplasmalogens. The degradation products of lysoplasmalogens, such as fatty aldehydes, are highly reactive electrophilic compounds that can form toxic adducts with cellular proteins and lipids. These interactions can lead to cellular dysfunction and contribute to various pathological conditions (58). Additionally, their accumulation in ischemic/reperfused tissues has been associated with cellular damage (59). However, we observed that the amount of lysoplasmalogens as a proportion of total plasmalogens in the liver of *Tmem86b* KO mice was only ∼3.5%, indicating that elevated lysoplasmalogens are rapidly converted into plasmalogens within the liver. Furthermore, in adipose tissue-specific *Tmem86a* KO mice, which also exhibited higher lysoplasmalogens, no toxic effects were observed. Instead, these mice showed elevated mitochondrial oxidative metabolism and energy expenditure, offering protection from high-fat diet-induced metabolic dysfunction (51). These findings suggest that any potential toxic effects of lysoplasmalogens are largely mitigated by their rapid reacylation into plasmalogens.

Interestingly, in both plasma and liver, we observed a more pronounced enrichment of PC(P) than PE(P) in *Tmem86b* KO mice. While lysoplasmalogens are known to be converted back into plasmalogens through CoA-independent transacylation in mammalian tissues, the precise molecular mechanisms of this process remain unclear. Our data suggest that the reacylation of LPC(P) to PC(P) is more complete than that of LPE(P) to PE(P), resulting in a larger effect on PC(P) (4-fold increase) compared to PE(P) (2-fold increase) in the liver of KO mice. Moreover, the primary enzyme responsible for the conversion of PE(P) to PC(P) in the liver, phosphatidylethanolamine N-methyltransferase (PEMT), is highly active in this organ (60). This suggests that elevated levels of PE(P) can directly result in an immediate increase in PC(P) levels in the liver (Fig. 1). Interestingly, despite a significant rise in hepatic plasmalogen content, the alkenyl chain composition remained unchanged in the liver of KO mice. Conversely, analysis of the acyl chain composition revealed a preferential incorporation of docosahexaenoic acid (DHA, 22:6) at the sn2 position of recycled plasmalogens in KO mice. The preferential incorporation of DHA at the sn2 position of recycled plasmalogens in KO mice may result from increased flux through the acyltransferase responsible for plasmalogen remodeling, leading to a higher proportion of DHA incorporation. Additionally, an increased availability of DHA in the tissue could further promote its incorporation. A previous study also demonstrated the selective targeting of DHA for incorporation into plasmalogens during de novo plasmalogen synthesis in RAW 264.7 cells (61). Apart from lysoplasmalogens and plasmalogens, ubiquinone levels were also increased in both plasma and liver of *Tmem86b* KO mice. This elevation in ubiquinone levels may result from antioxidant sparing. In the absence of efficient plasmalogen catabolism, plasmalogens accumulate and enhance antioxidant activity, reducing oxidative stress. This could decrease the demand for ubiquinone in reactive oxygen species (ROS) scavenging, leading to its accumulation. Further research is needed to elucidate this relationship. In addition to changes in plasma and liver plasmalogens, *Tmem86b* KO mice showed higher plasmalogen levels in NK cells while maintaining the alkenyl chain composition.

We also noted that the alkenyl and acyl chain compositions of plasma, liver, and cellular plasmalogens exhibited distinct characteristics. For instance, the relative proportion of plasmalogens with a P-16:0 alkenyl chain in PE(P) differs between the plasma, liver, and NK cells (∼40% in plasma, ∼47% in liver, and ∼56% in NK cells). Additionally, the relative proportion of PE(P) with 20:4 relative to 22:6 acyl chains is higher in the liver (∼54% and ∼25%, respectively), whereas the opposite is observed in plasma (20:4-containing PE(P): ∼32%; 22:6-containing PE(P): ∼50%). Intriguingly, the distinct alkenyl chain profiles of plasma, liver, and NK cell plasmalogens were precisely maintained in the enriched plasmalogen pools of *Tmem86b* KO mice. This suggests the presence of a regulatory mechanism in hepatocytes, and potentially other cell types, which ensures the preservation of this dynamic and conserved plasmalogen composition in cell membranes and secreted particles. The final steps of plasmalogen synthesis occur in the endoplasmic reticulum (ER), and following their synthesis, plasmalogens are transported to the Golgi apparatus for packaging, sorting, and transport to their final destinations (62). Lipoproteins are also assembled in the ER and undergo maturation in the Golgi apparatus in the liver (63). Therefore, it is possible that a quality control mechanism within the ER-Golgi network of the hepatocytes regulates the alkenyl chain composition of plasmalogens (defined in peroxisomes) for the plasma membrane and lipoprotein surface. This compositional homeostasis is maintained in *Tmem86b* KO mice despite elevated plasmalogen levels in plasma and tissues. However, the acyl composition appears to be preserved only in plasma (lipoproteins) and not in the liver or NK cells, indicating a tighter control over the composition of plasmalogens packaged into lipoprotein particles but leaving an imbalance in the liver itself. The increased remodeling of the acyl chain due to the downregulation of the degradation of the LPE(P) means that there is the opportunity to remodel with the available acyl chains, which are different in each tissue/cell. Furthermore, the alterations in the acyl chain composition could also result from the increased reacylation reactions in KO mice.

In contrast to the significant changes observed in plasma and liver plasmalogens, no notable differences in lysoplasmalogen or plasmalogen content at a class level were detected in the heart, brain, adipose tissues, or small intestine between *Tmem86b* KO and WT mice. The relatively higher plasmalogen levels in these tissues compared to the liver suggest slower catabolic rates, which is consistent with significantly lower *Tmem86b* expression in these organs, with the exception of the small intestine. This may account for the minimal impact of *Tmem86b* deletion on plasmalogen levels in these tissues. Interestingly, although *Tmem86b* expression is higher in the small intestine than in the liver, no significant changes in plasmalogen levels were observed in the small intestine of KO mice. We detected moderate *Tmem86a* expression in the small intestine of WT mice, relative to *Tmem86b* expression (Supplemental Fig. S2A) and found no significant difference in *Tmem86a* expression between WT and *Tmem86b* KO mice (Supplemental Fig. S2B). This suggests that the level of *Tmem86a* expression in the *Tmem86b* KO mice may be sufficient to maintain normal lysoplasmalogenase activity in the small intestine. Additionally, plasmalogen levels remained unchanged in the immune cells of *Tmem86b* KO mice, with the exception of NK cells. Higher expression of *Tmem86b* in NK cells could be a possible explanation for their selective plasmalogen enrichment in *Tmem86b* KO mice. If *Tmem86b* expression is higher in NK cells than in other immune cell types under normal conditions, its loss may disproportionately disrupt plasmalogen turnover in these cells, resulting in greater accumulation. This suggests that NK cells may have a higher intrinsic demand for plasmalogen remodeling or degradation compared to other immune cells.

Although the effects of *Tmem86b* ablation are mostly confined to the liver and plasma, the enriched hepatic and plasma lysoplasmalogen and plasmalogen pools in the KO mice could potentially support other tissues under conditions of plasmalogen deficiency. Lysoplasmalogens or plasmalogens derived from the liver may reach various tissues through lipoprotein-mediated transport and replenish the plasmalogen pool. For example, lysoplasmalogens may enter the brain via major facilitator superfamily domain-containing protein 2 (MFSD2A), a member of the major facilitator superfamily, and subsequently be converted into plasmalogens (64). However, the systemic distribution of liver-derived plasmalogens to other tissues remains a topic of debate. Further studies are needed to clarify these pathways.

To gain a more precise understanding of the role of hepatic TMEM86B in plasmalogen modulation, we also generated and characterized mice with hepatocyte-specific ablation of *Tmem86b* KO (HKO). The alterations in hepatic and plasma plasmalogen content and composition were consistent with those observed in global KO mice, reinforcing the liver's primary role in regulating the plasma plasmalogen pool. Given that the mouse strain used in this study has extremely low levels of low-density lipoprotein (LDL), it is likely that liver-derived lysoplasmalogens and/or plasmalogens are transported into the circulation via high-density lipoprotein (HDL) (65). Although the changes in liver and plasma plasmalogen levels between the global KO and HKO mice were similar, the increase in plasmalogen levels was slightly lower in the HKO mice compared to the global KO mice. For example, there was a 3-fold increase in hepatic PC(P) levels in HKO mice, compared to a 4-fold increase in the global KO mice. As the liver contains various cell types beyond hepatocytes, such as Kupffer cells, this difference is not unexpected, as *Tmem86b* expression is likely normal in cell types other than hepatocytes in the HKO mice.

There are also several limitations to this study. To minimize variability associated with hormonal fluctuations in females, which can significantly impact lipid metabolism, we exclusively used male mice. This approach enabled us to focus more consistently on the primary research question. However, we recognize the importance of sex as a biological variable and plan to explore potential sex-specific differences in future studies. Additionally, we characterized the *Tmem86b* KO mice at only one age (12 weeks). As such, the long-term, longitudinal effects of *Tmem86b* depletion remain unknown and warrant further investigation. Moreover, these mice may require exposure to pathogenic stimuli, such as a high fat diet or infection, to reveal any alterations in metabolic processes or antioxidant capacity.

In summary, this study demonstrates that global or hepatocyte-specific deletion of *Tmem86b* significantly elevates plasmalogen levels in the plasma and tissues of mice. This is the first instance of using a genetic approach to enhance plasmalogen levels in mammals, contrasting with previous models focused on their downregulation. Our findings provide valuable insights into plasmalogen biology, deepening our understanding of their role in cellular metabolism.

Plasmalogen modulation through dietary supplementation with plasmalogen precursors or intact plasmalogens has shown therapeutic benefits in various conditions, including obesity, atherosclerosis, fatty liver disease, and neurodegenerative disorders. In obesity, plasmalogen modulation found to reduce body weight, improve insulin sensitivity, and lower plasma lipid levels (66). Plasmalogen enrichment also demonstrated significant potential in reducing atherosclerotic plaques, along with exhibiting anti-inflammatory and antioxidant properties (67). In fatty liver disease, plasmalogen enrichment enhanced liver function by promoting fatty acid oxidation (68). Additionally, plasmalogen supplementation has been shown to improve hepatic steatosis by stimulating lipolysis in mice fed a high-fat, high-sucrose diet (69). Furthermore, plasmalogen enrichment demonstrated promising results in improving cognitive function in patients with mild Alzheimer's disease (70) and alleviating some clinical symptoms in patients with Parkinson's disease (71). These findings suggest that plasmalogen modulation for therapeutic purposes is generally safe, although the upper limit of plasmalogen levels associated with toxicity remains undefined. Further research is required to establish definitive safety thresholds for plasmalogen levels in clinical applications. Although dietary approaches of plasmalogen modulation have shown promising therapeutic benefits, targeting *Tmem86b* to modulate plasmalogen levels could serve as an alternative strategy-not only for elucidating the specific roles of plasmalogens in disease pathogenesis but also for developing novel therapeutic interventions.

## Data availability

The authors confirm that the data supporting the findings of this study are included in the article and the supplementary information. The raw data are available upon request from the corresponding author. The full oligonucleotide sequences of the primers and probes used for genotyping can be obtained upon reasonable request from Transnetyx (help@transnetyx.com).

## Supplemental data

This article contains supplemental data.

## Conflict of interest

The authors declare that they have no conflicts of interest with the contents of this article.
