# Supplementary material for: Modulation of endogenous plasmalogens by genetic ablation of lysoplasmalogenase (Tmem86b) in mice
Source: J Lipid Res. 2025 Apr 17;66(5):100808. doi: 10.1016/j.jlr.2025.100808 (PMC12144449; doi:10.1016/j.jlr.2025.100808)
Supplement: Supplemental Figures [file mmc1.docx]

**Supplemental Figure 1: Relative *Tmem86b* expression in different tissues of wild-type mice.**

mRNA expression levels of *Tmem86b* were normalised to *Hprt* expression and presented as mean±SEM (12 week old male mice, n=4-5/group, each circle represents individual mouse data) relative to liver. SI: small intestine, bAT: brown adipose tissue, sAT: subcutaneous adipose tissue.

**Supplemental Figure 2: Expression of *Tmem86b* and *Tmem86a* in the liver and small intestine of wild-type and *Tmem86b* knockout mice.**

mRNA expression levels were normalised to hypoxanthine phosphoribosyltransferase (*Hprt*) expression and presented as mean±SEM (12 week old male mice, n=4-5/group, each circle represents individual mouse data). In panel (A), expression levels are shown relative to *Tmem86b* expression in the specified tissue of wild-type mice. In panel (B), *Tmem86a* expression levels are shown relative to the wild-type group in the specified tissue. Statistical comparisons for each tissue were conducted using Student’s t-test; * indicates *P* <0.05.; *** indicates *P* <0.001. WT: wild-type; KO: homozygous *Tmem86b* knockout; SI: Small intestine.

**Supplemental Figure 3: *Tmem86b* expression in** **different tissues of** **hepatocyte-specific *Tmem86b* knockout mice.**

mRNA expression levels of *Tmem86b* were normalised to *Hprt* expression and presented as mean±SEM (12 week old male mice, n=4/group, each circle represents individual mouse data). The mean differences between the groups for a particular tissue were analysed using Student t test; ** indicates *P* <0.01. FC: floxed control; HKO: hepatocyte-specific *Tmem86b* knockout. SI: Small intestine.
